# Molecular Fingerprint and Dominant Environmental Factors of Nitrite-Dependent Anaerobic Methane-Oxidizing Bacteria in Sediments from the Yellow River Estuary, China

**DOI:** 10.1371/journal.pone.0137996

**Published:** 2015-09-14

**Authors:** Pengze Yan, Mingcong Li, Guangshan Wei, Han Li, Zheng Gao

**Affiliations:** State Key Laboratory of Crop Biology, College of Life Sciences, Shandong Agricultural University, Taian, Shandong, China; CAS, CHINA

## Abstract

Nitrite-dependent anaerobic methane oxidation (n-damo) is performed by “*Candidatus Methylomirabilis oxyfera*” (*M*. *oxyfera*), which connects the carbon and nitrogen global nutrient cycles. In the present study, *M*. *oxyfera*-like bacteria sequences were successfully recovered from Yellow River Estuary sediments using specific primers for 16S rRNA and *pmoA* genes. A *M*. *oxyfera*-like sequences analysis based on the 16S rRNA gene revealed greater diversity compared with the *pmoA* gene; the 16S rRNA gene sequences retrieved from the Yellow River Estuary sediments belong to groups A as well as B and were mainly found in freshwater habitats. Quantitative PCR showed that 16S rRNA gene abundance varied from 9.28±0.11×10^3^ to 2.10±0.13×10^5^ copies g^-1^ (dry weight), and the *pmoA* gene abundance ranged from 8.63±0.50×10^3^ to 1.83±0.18×10^5^ copies g^-1^ (dry weight). A correlation analysis showed that the total organic carbon (TOC) and ammonium (NH_4_^+^) as well as the ratio of total phosphorus to total nitrogen (TP/TN) influenced the *M*. *oxyfera*-like bacteria distribution in the Yellow River Estuary sediments. These findings will aid in understanding the n-damo bacterial distribution pattern as well as their correlation with surrounding environmental factors in temperate estuarine ecosystems.

## Introduction

Methane is the second most important greenhouse gas, and estimates suggest that it is responsible for 20% of global warming; the globe-warming potential caused by methane is 20-fold higher than carbon dioxide (CO_2_) on a centennial timescale [1,2]. In natural ecosystems, the major microbial oxidation processes for methane include aerobic methane oxidation and anaerobic methane oxidation (AMO); further, AMO coupled to sulfate reduction is performed by a combination of anaerobic methanotrophic archaea and sulfate-reducing bacteria [2,3]. Sulfate-dependent anaerobic oxidation is estimated to consume 80% of the methane from marine sediment [4]. However, our understanding of methane microbial oxidation greatly shifted upon discovering nitrite-dependent anaerobic methane oxidation (n-damo), which couples methane oxidation to nitrite reduction [5]. This finding demonstrates that oxygen may have been accessible to microbial metabolism before oxygenic photosynthesis evolved [6].

The n-damo process and targeted microorganisms were unclear until they were discovered in certain enrichment cultures [7–10]. The n-damo bacteria was identified as “*Candidatus Methylomirabilis oxyfera*” (*M*. *oxyfera*), which was affiliated with the candidate NC10 phylum based on the 16S rRNA gene sequence [7]. So both “*M*. *oxyfera*-like bacteria” and “n-damo bacteria” represent the same kind of bacteria. Thereafter, with suitable primer designs, multiple *M*. *oxyfera*-like bacteria sequences were successfully collected from different ecosystems using culture independent methods. For lake ecosystems, the existence of *M*. *oxyfera*-like bacteria was confirmed in sediments from Lake Constance, Lake Biwa, Qinghai-Tibetan saline lakes and West Lake [11–14]. In addition, both aerobic methane-oxidizing bacteria (MOB) and n-damo bacteria in in sediments of freshwater lakes on the Yunnan Plateau were investigated [15]. In contrast to the lake ecosystems, the n-damo bacteria exhibited relatively higher diversity in the Qiantang River and Jiaojiang Estuary sediments [16,17]. Moreover, the *M*. *oxyfera*-like bacteria sequences were detected in wetland ecosystems, such as paddy soil in northeast China, subtropical China and Yangtze River Plain; freshwater wetland in Xixi, Xiazhuhu; and coastal wetlands in Mai Po [18–22]. *M*. *oxyfera*-like bacteria have also been retrieved from sediments in the South China Sea, which confirmed that they are distributed in the continental shelves, slopes and abysses [23]. Further study has been conducted by investigating the vertical distribution of anaerobic methanotrophic archaea (ANME) and bacteria in a simple waste landfill in North China [24].

The Yellow River Estuary is located between the Laizhou Bay and Bohai Bay, and it is one of the three major estuaries in China. Each year, approximately 1.05×10^7^ tons of sediment is carried to and deposited in the estuary [25,26]. In addition, Shengli Oil Field, which is the second-largest oil field in China, is near the Yellow River Estuary. Recently, due to impact from rapid urbanization and anthropogenic activities, the nitrogen and organic matter loading have significantly increased in the estuary. Thus, the Yellow River Estuary was supposed to be an ideal habitat for the n-damo process. This is the first study that examines the existence of n-damo bacteria in a temperate estuarine ecosystem, and the unique geographical location of the Yellow River estuary gives the novelty of the current study. Moreover, previous studies suggest that concentrations of organic substances, ammonium (NH_4_^+^) and external oxygen as well as the molar ratio of ammonium to nitrate and nitrite were the main environmental factors that influenced n-damo bacteria distribution [16–18,20]. Will this pattern be observed in the Yellow River Estuary?

*M*. *oxyfera* can express the entire metabolic pathway of aerobic methane oxidation by particulate methane monooxygenase (pMMO). With the development of n-damo-specific PCR primers, which targeting alpha subunit of particulate methane monooxygenase pMMO (*pmoA* gene) and 16S rRNA gene, the molecular detection of *M*. *oxyfera*-like sequences became possible in a wide range of natural ecosystems. The objectives of the present study were to investigate the distribution, diversity and abundance of *M*. *oxyfera*-like bacteria in the Yellow River Estuary based on the 16S rRNA and particulate methane monooxygenase (*pmoA*) gene clone libraries as well as quantitative PCR and to examine the relationships between *M*. *oxyfera*-like bacteria distribution and estuarine environmental factors.

## Materials and Methods

### Sample Collection and Physicochemical Analysis

The Yellow River Estuary, which is the confluence of the Yellow River, Bohai Bay and Laizhou Bay, lies in an eastern coastal area in Dongying City, Shandong Province, China. Every year, approximately 1.05×10^7^ tons of sediment deposit in the estuary [25,26]. Sediment samples (about 0–5 cm) were collected in triplicate from five representative sites in the Yellow River Estuary in October 2012. All sediments were obtained from public land, for which no permissions were required for sampling, and no impacts on endangered species. Sampling site A (sample SA) (37°46′25.75″N, 119°20′21.24″E) includes the seawater and is located relatively distal to mainland; sampling sites B (sample SB) (37°50′44.1″N, 119°16′47.34″E) and C (sample SC) (37°48′37.90″N, 119°17′12.39″E) are located in the confluence of the Yellow River and Bohai Bay; sampling site D (sample SD) (N37°43′58.12″N, E119°18′28.60″E) is near the mainland and located adjacent to an oil field; and sampling site E (sample SE) (37°45′38.44″N, 119°9′43.4″E) includes river water and is located near the estuary. The five samples in this study can be subdivided into two types, three belonged to the area of tidal flats (SA, SC and SD) and two located in the confluence of the river (SB and SE).

All sediment samples were placed into ice boxes immediately and transported back to the laboratory shortly after collection. The samples used for molecular studies were stored at –80°C, and the samples used for physicochemical analysis were immediately processed. The *in situ* pH, salinity (Sal) and dissolved oxygen (DO) of the bottom water (BW) above the sediment samples were measured using portable instruments (Leici, Shanghai, China) at the site. The sediment pH was measured after the sediments were mixed with deionized water free of CO_2_ at the ratio (sediment/water) 1:2.5 using portable instruments (Leici, Shanghai, China). Total nitrogen (TN), total phosphorus (TP) and total organic carbon (TOC) concentrations were measured according to the methods described in previous study (Sun et al. 2013a). The ammonium (NH_4_^+^) and nitrate (NO_3_^–^) in the sediment samples were treated in accordance with Xia et al [27]; their concentrations were then determined using an AutoAnalyzer3 (Bran+Luebbe, Hamburg, Germany). The dry weight was determined after drying the sediment samples for 24 h at 110°C. The physicochemical characteristics of the sediment samples used in this study are listed in S1 Table.

### DNA Extraction, PCR Amplification

The genomic DNA from each sample was extracted in triplicate using the E.N.Z.A.^TM^ soil DNA Kit (Omega, GA, USA) in accordance with the manufacturer’s instruction. The quality of the extracted DNA was examined using electrophoresis and a 1.0% agarose gel. The extracted DNA was stored at –20°C for further analysis.

Different pairs of primers were used to amplify the *M*. *oxyfera*-like bacteria 16S rRNA and *pmoA* genes. The *M*. *oxyfera*-like bacteria 16S rRNA gene was amplified using the primer set 202F-1545R [28] followed by the primer set qp1F-qp2R [7]. The *pmoA* gene was amplified using the primer set A189_b-cmo682 followed by the primer set cmo182-cmo568 [29]. The prime sequences and detailed thermal profiles are shown in S2 Table.

### Cloning and Sequencing

The PCR products were ligated into the pMD18-T vector (TaKaRa, Dalian, China) then transformed into *E*.*coli* DH5α competent cells (TaKaRa, Dalian, China); ten gene libraries total were constructed. A maximum of about 40 positive clones were randomly selected for sequencing by Majorbio Biomedical Technology Co. Ltd (Shanghai, China). The sequence quality was verified using the Chromas (Version 2.22) program, and UCHIME was used to remove the chimeras [30].

### Phylogenetic Analysis

A BLAST search was performed, and reference sequences were retrieved from GenBank (http://www.ncbi.nlm.nih.gov/GenBank/). The sequences were aligned using MEGA 5.1 software [31] and then manually verified as well as trimmed. Phylogenetic trees were constructed using the neighbor-joining (NJ) method based on the nucleotide sequences [31], and the confidence levels to the tree nodes were examined using a bootstrap analysis with 1,000 replicates.

### Quantitative PCR

The abundance of *M*. *oxyfera*-like bacteria was estimated by assessing the 16S rRNA and *pmoA* gene copy numbers using the primer pairs qp1F-qp1R and cmo182-cmo568, respectively (S2 Table). Both assessments were followed by the amplification processes described in S2 Table. The plasmid concentrations were determined through spectrophotometric measurements using a ND-2000 UV Spectrophotometer (NanoDrop, DE, USA). We performed qPCR to generate standard curves, and the samples were analyzed using a CFX96TM Real-time System (Bio-Rad, CA, USA). The amplification reactions were performed using SYBR Premix Ex Taq (TaKaRa, Dalian, China) in a total volume of 20 μl. Each reaction mixture contained approximately 10 ng of template DNA, 10 μl of 2×SYBR Premix Ex Taq, and 0.15 μl of each primer (10 μM). All samples were analyzed in triplicate. The standard curves were obtained using serial dilutions of a known number of plasmid copies containing a target gene fragment; the R^2^ values from both qPCR experiments were greater than 0.993.

### Statistical Analysis

Mothur [32] was used to analyze the operational taxonomic units (OTUs) by defining a 3% cut-off for the 16S rRNA gene and a 7% cut-off for the *pmoA* gene. CANOCO for Windows software (version. 4.5) was used to identify the relationships between *M*. *oxyfera*-like bacterial communities and environmental variables using a redundancy analysis (RDA). Pearson correlations analysis between the *M*. *oxyfera*-like bacterial diversity, abundance and environmental factors were performed using SPSS (Statistical Package for the Social Sciences) 19 software (Chicago, Illinois, USA). The diversity index, rarefaction analysis and principal coordinate analysis (PCoA) were calculated using PAleontological STatistics (PAST) software (version. 2.17) [33].

### Nucleotide Sequence Accession Numbers

The nucleotide sequences reported in this study were deposited in the GenBank database under the accession numbers KP296952-KP297113 (*M*. *oxyfera* 16S rRNA) and KP297114-KP297241 (*M*. *oxyfera pmoA*).

## Results

### Environmental Characteristics of the Sediment Samples

The physicochemical characteristics of the Yellow River Estuary samples are shown in S1 Table. The sediment samples included a relatively high total nitrogen (TN) concentration, varying from 816 to 1,391 mg kg^-1^dry weight. The total organic carbon (TOC) differed greatly among five sampling sites, varying from 1305 mg kg^-1^ to 6053 mg kg^-1^. The samples included high ammonium (NH_4_^+^) concentration; the highest ammonium concentration (27.74 mg kg^-1^) was detected in sample SC. Simultaneously, the bottom water (BW) pH and dissolved oxygen (DO) were also determined, ranging from 7.9 to 8.25 and 6.01 to 8.52 mg L^-1^. Notably, due to the great slits carrying volume of Yellow River, most of our samples are riverine, however the bottom water salinity exhibited extremely high concentration (four out of five samples are above 20‰), which contributed to an unique habitat for the n-damo bacteria.

### Phylogenetic Diversity of *M*. *oxyfera*-Like Bacterial 16S rRNA Genes

**Fig 1:**
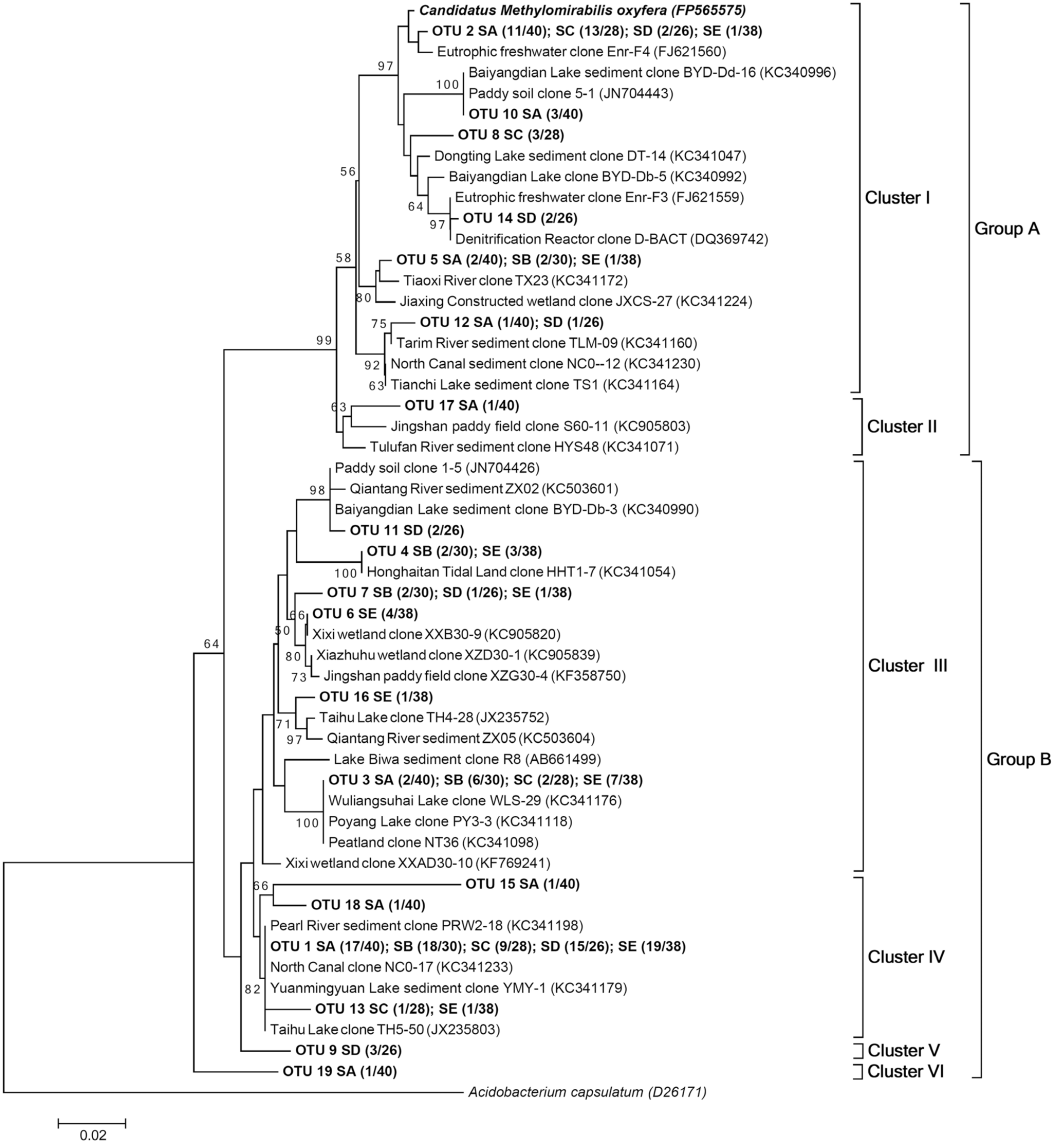
A neighbor-joining phylogenetic tree showing the phylogenetic affiliations of *M*. *oxyfera*-like bacteria 16S rRNA gene sequences from the Yellow River Estuary. The bootstrap values were based on 1,000 replicates, the *scale bar* represents 2% sequence divergence, and only >50% values are shown. In brackets, the numbers preceding the slash represent the numbers of clones that belong to the OTU, and the numbers following the slash represent the total numbers of clones in corresponding clone libraries.

The bacterial 16S rRNA gene sequences affiliated with the NC10 phylum were successfully amplified from the Yellow River Estuary sediment samples. Through a nested PCR approach, we acquired 162 n-damo bacteria-related 16S rRNA gene sequences from five samples with 92.1% to 100% coverage. These sequences showed 89.32–99.13% identity to the *Candidatus Mtheylomirabilis oxyfera* 16S rRNA gene (FP565575). Mothur analysis indicated that all of the 16S rRNA gene sequences from the sampling sites contained 19 OTUs with a 3% cut-off value. Furthermore, phylogenetic analysis showed that the 19 OTUs could be categorized into 2 main *M*. *oxyfera*-like bacteria groups, namely groups A and B, according to Shen et al. [34] (Fig 1). Group A contains 2 clusters (I and II); all clones belonging to group A exhibited a high sequence similarity to the *Candidatus Methylomirabilis oxyfera* (FP565575), which exhibited identity from 95.42% to 99.13%. However, group B, which accounted for 73.5% of the n-damo 16S rRNA sequences collected, was divided into 4 clusters (III, IV, V and VI), the sequences for which exhibited an identity to the *Candidatus Methylomirabilis oxyfera* (FP565575) that ranged from 89.32% to 93.91%. Cluster IV, which included almost half (49.5%) of the 16S rRNA sequences collected, was the largest cluster. The OTU1 in cluster IV that belonged to group B was the most abundant taxon, and the OTU1 sequences were successfully retrieved from each samples and accounted for approximately 48.1% of the 16S rRNA gene sequences collected. OTU1 was closely related to the sequences recovered from Pearl River sediment and the North Canal [35]. OTU2 fell into group A and was the second largest OTU, the sequences for which were successfully obtained from samples except SB; OTU2 was responsible for approximately 27.8% of the total 16S rRNA gene sequences. This OTU was closely related to the clones recovered from eutrophic freshwater [7]. The remaining OTUs were relatively fewer than OTU1 and OTU2. The sequences from sample SA were divided into 5 different clusters, which exhibited a more extensive distribution. However, the sequences from samples SB, SC and SE were divided into 3 clusters, which exhibited a relatively intensive distribution. The *M*. *oxyfera*-like bacterial 16S rRNA gene diversity in each sample was determined using rarefaction analysis (S1a Fig). Diversity indices, such as the number of OTUs, Shannon-Wiener index, Evenness index and S_chao1_, were also used to indicate the *M*. *oxyfera*-like bacteria 16S rRNA gene diversity. The OTU number, S_chao1_, Evenness index and Shannon-Wiener index ranged from 4 to 8, 5.00 to 19.00, 0.5331 to 0.7107 and 1.170 to 1.674, respectively (based on a 97% cutoff, Table 1). Among the five sediment samples, sample SA represents the highest 16S rRNA diversity, sample SB, SC showed the lowest 16S rRNA diversity. Sample SD, SE exhibited an intermediate level of 16S rRNA diversity.

**Table 1:** Diversity of n-damo bacteria in the sediment samples (based on percentage sequence identify of 97% similarity for 16S rRNA genes and 93% for *pmoA* genes).

Sample	Number of sequences		Number of OTUs		Coverage		Shannon		Evenness		Chao1	
	16S	*pmoA*	16S	*pmoA*	16S	*pmoA*	16S	*pmoA*	16S	*pmoA*	16S	*pmoA*
SA	40	28	8	4	0.925	0.964	1.674	1.234	0.5331	0.6780	13.33	5.0
SB	30	18	5	4	1.000	0.889	1.170	1.226	0.6444	0.6816	5.00	5.5
SC	28	28	4	8	1.000	0.920	1.268	1.887	0.7107	0.8253	5.00	8.5
SD	26	28	7	3	0.923	1.000	1.409	0.977	0.5846	0.8856	7.25	3.0
SE	38	26	7	1	0.921	1.000	1.574	0.000	0.5363	1.0000	19.00	1.0

### Phylogenetic Diversity of *M*. *oxyfera*-Like Bacterial *pmoA* Genes

**Fig 2:**
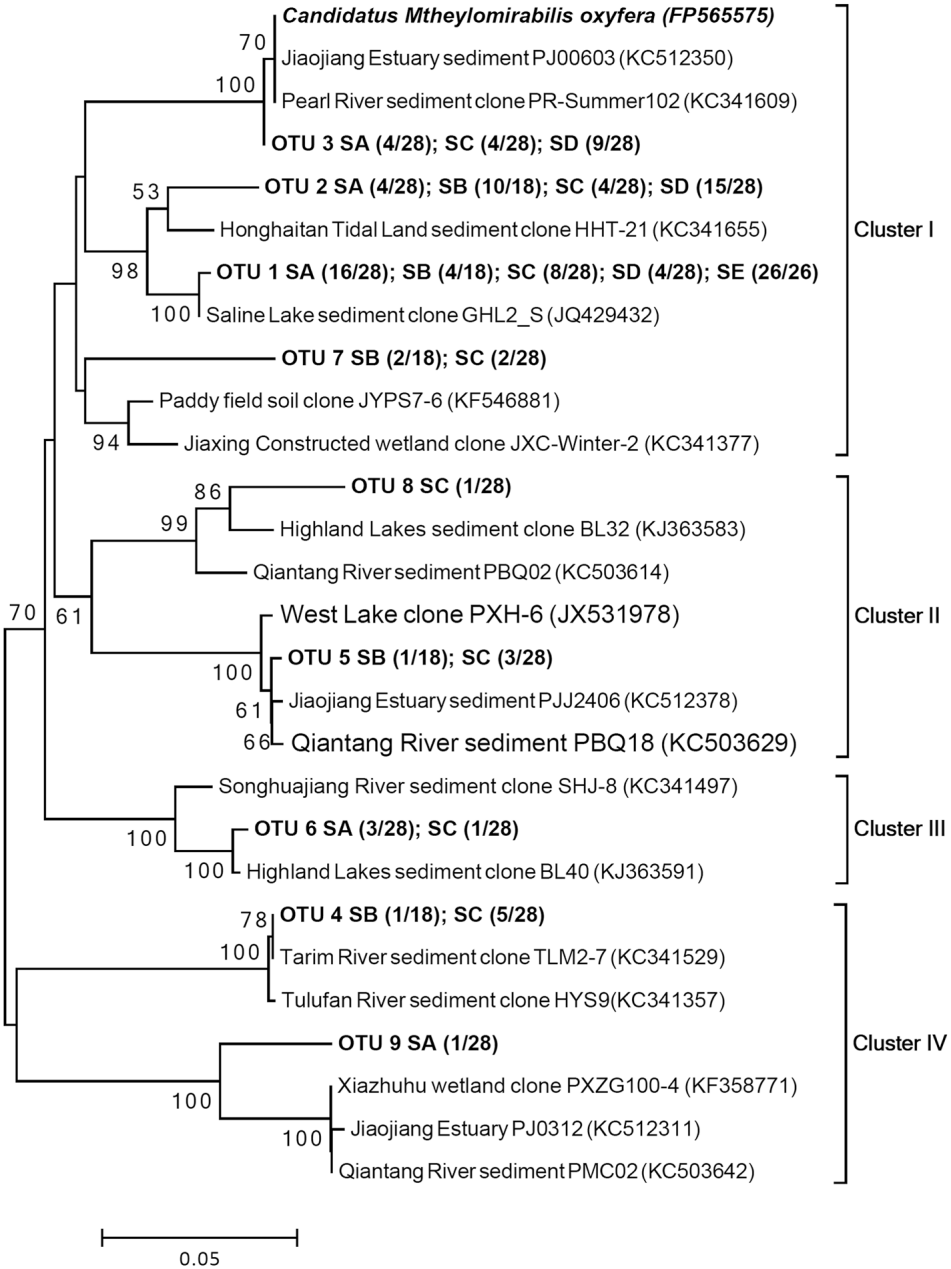
A neighbor-joining phylogenetic tree showing the phylogenetic affiliations of *M*. *oxyfera*-like bacteria *pmoA* gene sequences from the Yellow River Estuary. The bootstrap values were based on 1,000 replicates, the scale bar represents 10% sequence divergence, and only >50% values are shown. In brackets, the numbers preceding the slash represent the number of clones belong to the OTU, and the numbers following the slash represent the total number of clones in corresponding clone libraries.

Using a nested PCR approach, we acquired 128 *pmoA* gene sequences from five samples with 88.9% to 100% coverage. The data indicate that the *M*. *oxyfera* bacteria *pmoA* genes from the Yellow River Estuary sediments were efficiently covered. These sequences exhibit 86.63% to 99.74% identity to the *Candidatus Methylomirabilis oxyfera pmoA* gene (FP565575) at the nucleotide level. Nine total OTUs were obtained by clustering *pmoA* gene sequences using a 7% threshold. Phylogenetic analysis showed that the 9 OTUs could be categorized into four clusters related to *M*. *oxyfera* (Fig 2). Cluster I was the dominant cluster; the cluster I sequences were from the sampling sites and accounted for 87.5% of the *pmoA* sequences collected. Further, the cluster I sequences were closely related to clones from Jiaojiang Estuary, Pearl River and Honghaitan Tidal Land sediments [17,35], which exhibited 90.75% to 99.74% similarity to the *M*. *oxyfera pmoA* gene. The cluster II sequences were only obtained from the SB and SC samples; these sequences exhibited 87.40% to 90.49% identity to *M*. *oxyfera*. This cluster also includes reference sequences from Jiaojiang Estuary, Qiantang River and West Lake sediments [10,16,17]. Only 1 OTU (OTU6) from the Yellow River Estuary branched into the smallest cluster (cluster III); the OTU6 sequences were discovered in the SA and SC samples and exhibited 89.72% identity to the *M*. *oxyfera pmoA* gene. These sequences were closely related to sequences from Songhuajiang River and highland lake sediments [35]. Moreover, cluster IV was the second largest cluster, the sequences from which were detected in the SA, SB and SC samples, which exhibited 86.63% to 87.66% similarity to the *M*. *oxyfera pmoA* gene. The closely related reference sequences in cluster IV were mainly obtained from Jiaojiang Estuary, Qiantang River, Tarim River and Xiazhuhu wetland sediments [16,17,35,36]. In addition, the SC sample was the only sample that exhibited sequences in the four clusters. In contrast, sample SE only included 1 OTU from cluster I; thus, it exhibited the least diversity. The diversity of the *M*. *oxyfera*-like bacterial *pmoA* genes for each sample was compared using rarefaction analysis (S1b Fig). To investigate *M*. *oxyfera*-like bacteria diversity at different sampling sites, diversity indices, such as the number of OTUs, Shannon-Wiener index, Evenness index and S_chao1_, were calculated for each representative sample and are listed in Table 1. The OTU number, S_chao1_, Evenness index and Shannon-Wiener diversity index ranged from 1 to 8, 1.0 to 8.5, 0.6780 to 1.0000 and 0.000 to 1.887, respectively (based on a 93% cutoff, Table 1). However, the *pmoA* gene diversity observed in each sample was different to the diversity of 16S rRNA gene. Among the five sediment samples, sample SA represents the highest *pmoA* diversity, sample SB, SC showed the lowest *pmoA* diversity. Sample SD, SE exhibited a intermediate level of *pmoA* diversity. This might be due to the fact that the 16S rRNA gene and *pmoA* gene similarity levels are different, 97% for 16S rRNA gene and 93% for *pmoA* gene. In addition, the sensitivities and specificities of primers might also be different.

### Quantitative Analysis of *M*. *oxyfera*-Like Bacterial 16S rRNA and *pmoA* Genes

**Fig 3:**
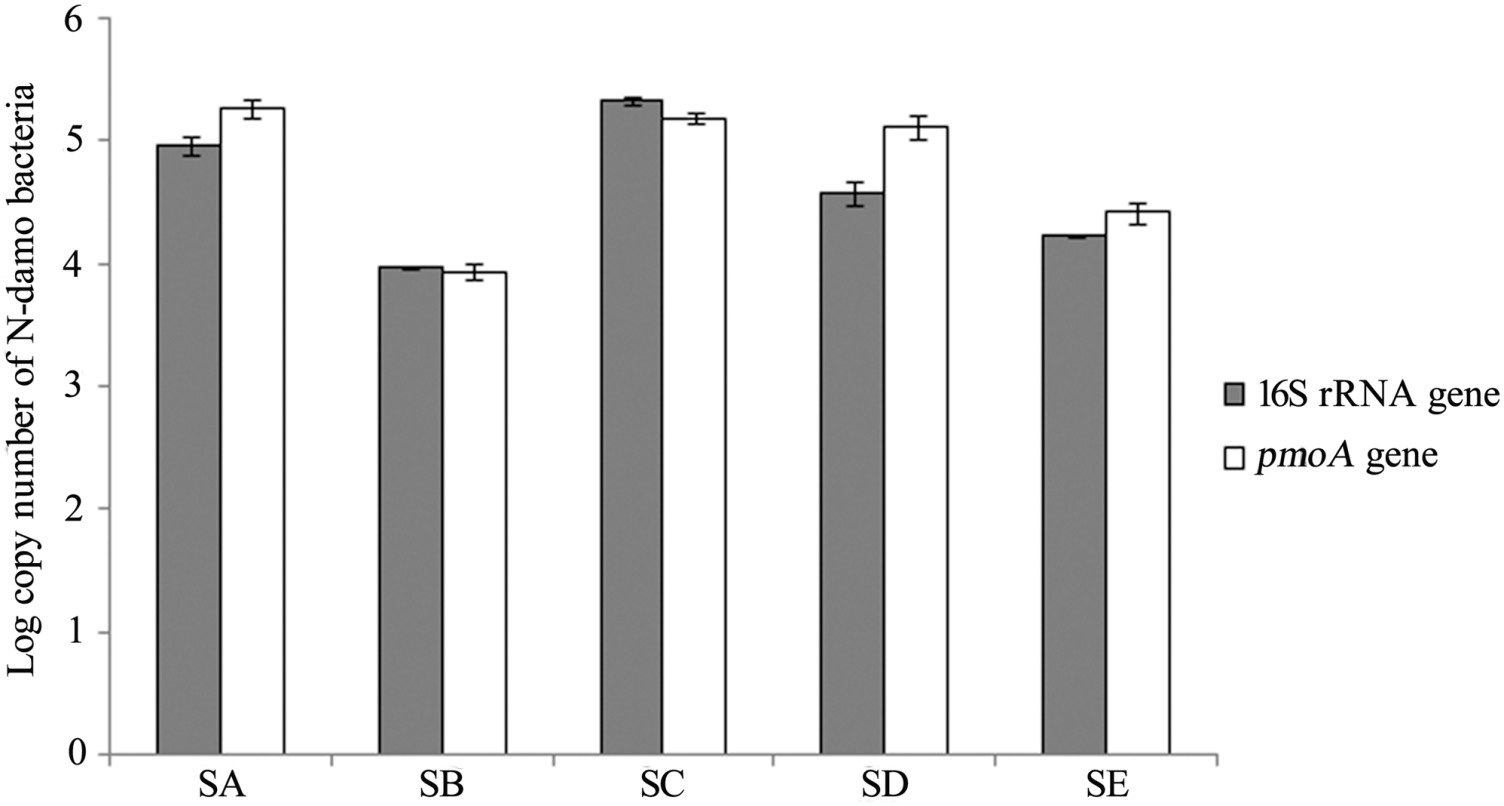
Log copy numbers of the *M*. *oxyfera*-like bacteria 16S rRNA and *pmoA* genes in five samples from the Yellow River Estuary sediments.

The abundance of *M*. *oxyfera*-like bacteria in the five samples was estimated using the bacterial 16S rRNA and *pmoA* gene copy numbers through qPCR. The *M*. *oxyfera*-like bacteria 16S rRNA gene copy numbers ranged from 9.28±0.11×10^3^ to 2.10±0.13×10^5^ copies g^-1^ (dry weight), and the *M*. *oxyfera*-like bacteria *pmoA* gene copy numbers varied significantly from 8.63±0.50×10^3^ to 1.83±0.18×10^5^ copies g^-1^ (dry weight) (Fig 3). The *M*. *oxyfera*-like bacterial 16S rRNA and *pmoA* gene copy numbers varied from different samples. Both qPCR results showed that the *M*. *oxyfera*-like bacterial abundance in samples SB and SE was lower than in the other samples; samples SA and SC showed relatively higher copy numbers with approximately 10^5^ copies g^-1^ (dry weight). The remaining sample SD presented moderate copy numbers at 10^4^ copies g^-1^ (dry weight).

### Correlations between *M*. *oxyfera*-Like Bacterial Distribution and Environmental Factors

A principal coordinate analysis (PCoA) was used to estimate the similarity of the *M*. *oxyfera*-like bacteria community composition based on the OTUs. Based on the 16S rRNA gene sequence PCoAs, *M*. *oxyfera*-like bacterial communities were divided into three groups; samples SA and SC shared similar community compositions, and SB and SE exhibited similar community compositions. However, sample SD was not closely related to the above two groups (S2a Fig). We generated different classification results for the PCoAs based on the *pmoA* gene sequences. The *M*. *oxyfera*-like bacterial communities were classified into four groups based on their *pmoA* gene sequences. The SB and SD samples shared similar community compositions. Notably, SA, SC and SE were not closely related and formed three independent groups (S2b Fig).

**Fig 4:**
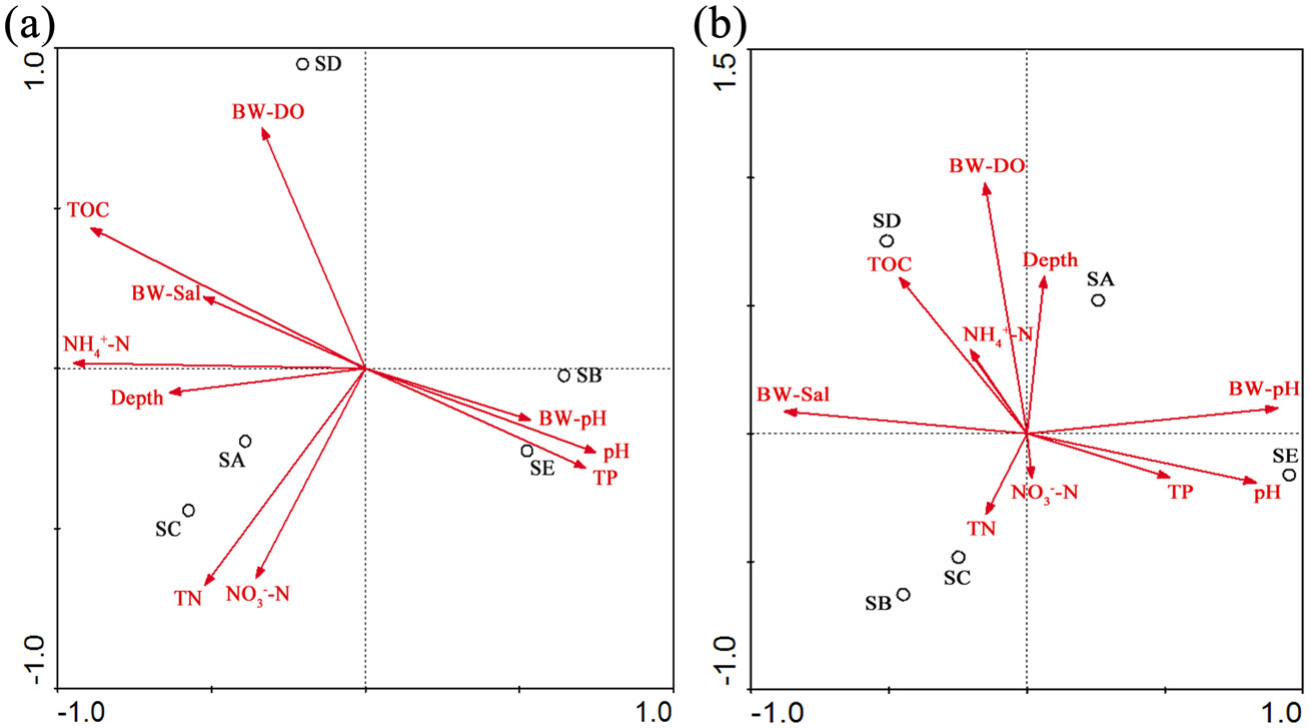
RDA ordination plots show the correlation between the *M*. *oxyfera*-like bacteria community structure and environmental factors using the 16S rRNA (a) and *pmoA* (b) gene sequences from Yellow River Estuary sediments. The correlations between the environmental factors and RDA axes are represented by the length and angle of the *arrows*. Depth: depth of the overlying water, TN: total nitrogen, TP: total phosphorus, NH_4_^+^-N: ammonium, NO_3_^−^-N: nitrate, TOC: total organic carbon, BW: bottom water, Sal: salinity, and DO: dissolved oxygen.

Detrended correspondence analysis (DCA) showed that the maximum gradient lengths were 1.570 and 1.666 for 16S rRNA and *pmoA*, respectively. We used RDA to further discern the relationship between the *M*. *oxyfera*-like bacterial distribution and environmental factors. The RDA results showed that the 16S rRNA and *pmoA* gene sequence axes could explain 76.7% and 79.1% of the cumulative percentage variances in the relationship between the *M*. *oxyfera*-like bacterial distribution and environment factors. Based on 16S rRNA gene diversity, the RDA results showed that the *M*. *oxyfera*-like bacterial distribution significantly correlated with NH_4_^+^–N and TOC (P<0.05, 1,000 Monte Carlo permutations) (Fig 4). However, the bottom water pH and bottom water salinity showed a significant correlation with *pmoA* gene diversity (P<0.05, 1,000 Monte Carlo permutations).

Moreover, Pearson moment correlation analysis demonstrated that bottom water pH, bottom water salinity, pH and NH_4_^+^–N significantly correlated with the *pmoA* Shannon-Wiener index (P<0.05). The TP/TN significantly correlated with the number of *pmoA* OTUs, Shannon-Wiener index, and S_chao1_ (P<0.05). The 16S rRNA gene abundance and S_chao1_ strongly correlated with the TP/TN and bottom water pH, respectively (Table 2).

**Table 2:** Pearson correlation analysis of environmental factors and *M*. *oxyfera*-like bacteria diversity and abundance in sediments of Yellow River Estuary.

Environmental factors	Number of OTUs		Shannon		Chao1		Abundance	
	16S	*pmoA*	16S	*pmoA*	16S	*pmoA*	16S	*pmoA*
Depth	0.562	0.152	0.636	0.295	0.156	0.184	0.292	0.803
BW-pH	0.583	-0.829	0.643	*-0*.*942* *	*0*.*948* *	-0.843	-0.548	-0.437
BW-Sal	-0.337	0.699	-0.465	*0*.*896* *	-0.859	0.755	0.411	0.509
BW-DO	0.761	-0.363	0.515	-0.139	0.12	-0.397	-0.272	0.501
NO_3_^−^-N	-0.055	0.508	0.412	0.554	-0.091	0.623	0.469	0.424
NH_4_^+^-N	-0.138	0.633	0.112	0.545	-0.243	0.473	0.84	0.852
TN	-0.359	0.785	-0.09	0.765	-0.316	0.849	0.736	0.497
TP	0.408	-0.608	0.313	-0.539	0.542	-0.441	-0.656	-0.549
TP/TN	0.486	*-0*.*948* *	0.299	*-0*.*941* *	0.659	*-0*.*900* *	*-0*.*879* *	-0.738
TOC	0.053	0.46	0.083	0.517	-0.376	0.327	0.563	0.856
pH	0.354	-0.779	0.389	*-0*.*903* *	0.818	-0.753	-0.611	-0.685

*p<0.05

**p<0.01, respectively, as determined by SPSS 19 software (Chicago, Illinois, USA). *Depth*: depth of the overlying water, *BW*: bottom water, *Sal*: Salinity, *DO*: dissolved oxygen, *NH*_*4*_^+^ *-N*: ammonium, *NO*_*3*_^—^ *N*: nitrate, *TN*: total nitrogen, *TP*: total phosphorus, *TOC*: total organic carbon

## Discussion

A previous study confirmed *M*. *oxyfera*-like bacteria in Jiaojiang Estuary sediments [17], and a high level of nitrate also suggests that the Yellow River Estuary might be an ideal habitat for n-damo processes. However, in this study, the clone library results clearly confirm *M*. *oxyfera*-like bacteria in the Yellow River Estuary. Quantitative PCR results further confirm the existence of *M*. *oxyfera*-like bacteria. This is the first study that has examined the presence of n-damo bacteria in a temperate estuarine ecosystem.

The 16S rRNA gene sequences in the *M*. *oxyfere* genome were obtained from all of the Yellow River Estuary samples. In this study, at least 4 OTUs were found in a single sample, and 7 or 8 OTUs have been observed in samples SA, SD and SE (Fig 1). Most previous studies were based on detecting the *M*. *oxyfera*-like 16S rRNA genes that exhibit lower diversity than in the Yellow River Estuary. For instance, Deutzmann and Schink [11] examined *M*. *oxyfera*-like bacteria diversity from the profundal and littoral sediments of Lake Constance, and they detected three OTUs and five OTUs, respectively. Kojima et al. [12] observed six OTUs in the profundal sediments of Lake Biwa. Fifteen and three *M*. *oxyfera*-like bacterial OTUs were discovered in the Qiantang River and Jiaojiang Estuary sediments, respectively [16,17]. Therefore, the *M*. *oxyfera*-like bacteria 16S rRNA gene diversity in the Yellow River Estuary was relatively higher than most other habitats that have been previously examined. Through identifying *M*. *oxyfera*-like bacteria on functional gene level, a similar trend was obtained. In this study, only 1 OTU was detected in sample SE, and at least 3 OTUs were observed in the remaining 4 samples (Fig 2). For example, a previous study reported low diversities in Lake Constance [11] and Lake Biwa [12] each with 1 and 2 OTUs. Moreover, a total of 13 and 16 OTUs were detected in the Qiantang River [16] and Jiaojiang Estuary sediments [17], respectively. In addition, based on the results of phylogenetic analysis, we found a heterogeneous distribution of *M*. *oxyfera*-like bacteria was observed in our study, 94.1% sequences of all (64/68) retrieved from sample SB and SE were categorized into group B, while sequences retrieved from three tidal flats were roughly equally categorized into group A (41.5%) and B (58.5%).

A previous study showed that groups A and B were dominated by freshwater n-damo 16S rRNA gene sequences; whereas groups D and E were mainly composed of marine n-damo 16S rRNA gene sequences [23]. In this study, the 16S rRNA gene sequences from the Yellow River Estuary sediments were affiliated with groups A and B, which indicated that the n-damo bacteria community structure in the estuary was dominated by freshwater types. In addition, the community composition was similar to the n-damo bacteria in two coastal regions [17,35], from which all of the sequences were divided into groups A and B. However, this type of community structure differs from the Mai Po coastal area, the sequences from which were grouped with both freshwater and marine sequences [18].What contributions may be due to the fact that several billion tons of freshwater sediment that deposits in the estuary every year and most of the samples were from the Yellow River silts. Notably, that the high bottom water salinity demonstrates a possibility that these freshwater ecotypes survive the high salinity, previous research put forward the same prediction [37].

In this study, both the 16S rRNA and *pmoA* genes were used to test the *M*. *oxyfera*-like bacterial abundance and similar overall trends were observed (Fig 3). Specifically, the primer set cmo182-cmo568 was used to evaluate the *pmoA* gene abundance, which further accurately examined the n-damo bacteria abundance. The *M*. *oxyfera*-like bacteria abundance demonstrated by both the 16S rRNA and *pmoA* genes in the Yellow River Estuary ranged from 10^3^–10^5^ copies g^-1^ (dry weight). Compared with other studies, the abundance of the *M*. *oxyfera*-like bacteria of the Yellow River Estuary that we observed was lower than in the Jiaojiang Estuary [17], Mai Po wetland [18] and Xixi wetland [21] with 5.80×10^4^–8.35×10^7^, 2.65×10^5^–6.71×10^5^ and 1.70×10^6^–1.0×10^7^ copies g^-1^ (dry weight), respectively. However, the n-damo bacteria abundance in the Yellow River Estuary was slightly higher than in the paddy soil-JN with 6.5×10^3^–1.0×10^5^ copies g^-1^ (dry weight) [20].

The environmental characteristics are important factors that influence the *M*. *oxyfera*-like bacteria distribution and diversity. In this study, the RDA result based on the 16S rRNA gene showed that the TOC and NH_4_^+^-N significantly correlated with the *M*. *oxyfera*-like bacteria community structure and distribution, which are similar to previous studies on the Qiantang River [16] and Mai Po wetland sediments [18]. In addition, Pearson correlation analysis indicates that the TP/TN also strongly influenced the *M*. *oxyfera*-like bacteria distribution in the Yellow River Estuary sediments (Table 2). However, according to the RDA results using the 16S rRNA and *pmoA* genes, different environmental factors correlate with the n-damo bacteria distribution, which was also reported in a previous study on the Mai Po wetland [18]. It is speculated that the environmental factors that influence the n-damo bacteria distribution and community structure are multiple and complex. What’s more, riverine sediments and high salinity combination may contribute to an unique habitat for the n-damo bacteria, this may be the reason for higher diversity and lower abundance of *M*. *oxyfera*-like bacteria.

## Conclusions

In summary, this study shows the *M*. *oxyfera*-like bacteria diversity and distribution in a temperate estuarine ecosystem for the first time, which further expands our knowledge of *M*. *oxyfera*-like bacteria distribution in natural ecosystems. Based on phylogenetic analysis of the 16S rRNA and *pmoA* genes, it is suggested that the *M*. *oxyfera*-like bacteria diversity is relatively higher than many other ecosystems. The *M*. *oxyfera*-like bacteria abundance was detected using both the 16S rRNA and *pmoA* genes, which strongly confirm *M*. *oxyfera*-like bacteria in the Yellow River Estuary. In addition, the TOC, NH_4_^+^and TP/TN concentrations influenced the *M*. *oxyfera*-like bacteria distribution in the estuarine sediments. Future studies should focus on the measurement of the gene expression of n-damo bacteria, potential rates of the n-damo process and the temporal distribution of *M*. *oxyfera*-like bacteria.

## Supporting Information

S1 Fig Rarefaction analysis of n-damo bacteria communities based on amplified 16S rRNA (a) and *pmoA* (b) gene sequences in the sediments of Yellow River Estuary. Mothur was used with 3% or 7% nucleotide sequence variation for OTU determination for 16S rRNA or *pmoA* gene, respectively.
(TIF)

S2 Fig PCoA ordination diagram of the *M*. *oxyfera*-like communities calculated with 16S rRNA gene sequences (a) and *pmoA* gene sequences (b) in the sediments of Yellow River Estuary. (TIF)

S1 Table Physicochemical characteristics of the sediment samples used in present study. (DOCX)

S2 Table The primers and thermal profiles used in this study. (DOCX)
