# Supplementary figures and images for: Molecular Fingerprint and Dominant Environmental Factors of Nitrite-Dependent Anaerobic Methane-Oxidizing Bacteria in Sediments from the Yellow River Estuary, China
Source: PLoS One. 2015 Sep 14;10(9):e0137996. doi: 10.1371/journal.pone.0137996 (PMC4569144; doi:10.1371/journal.pone.0137996)

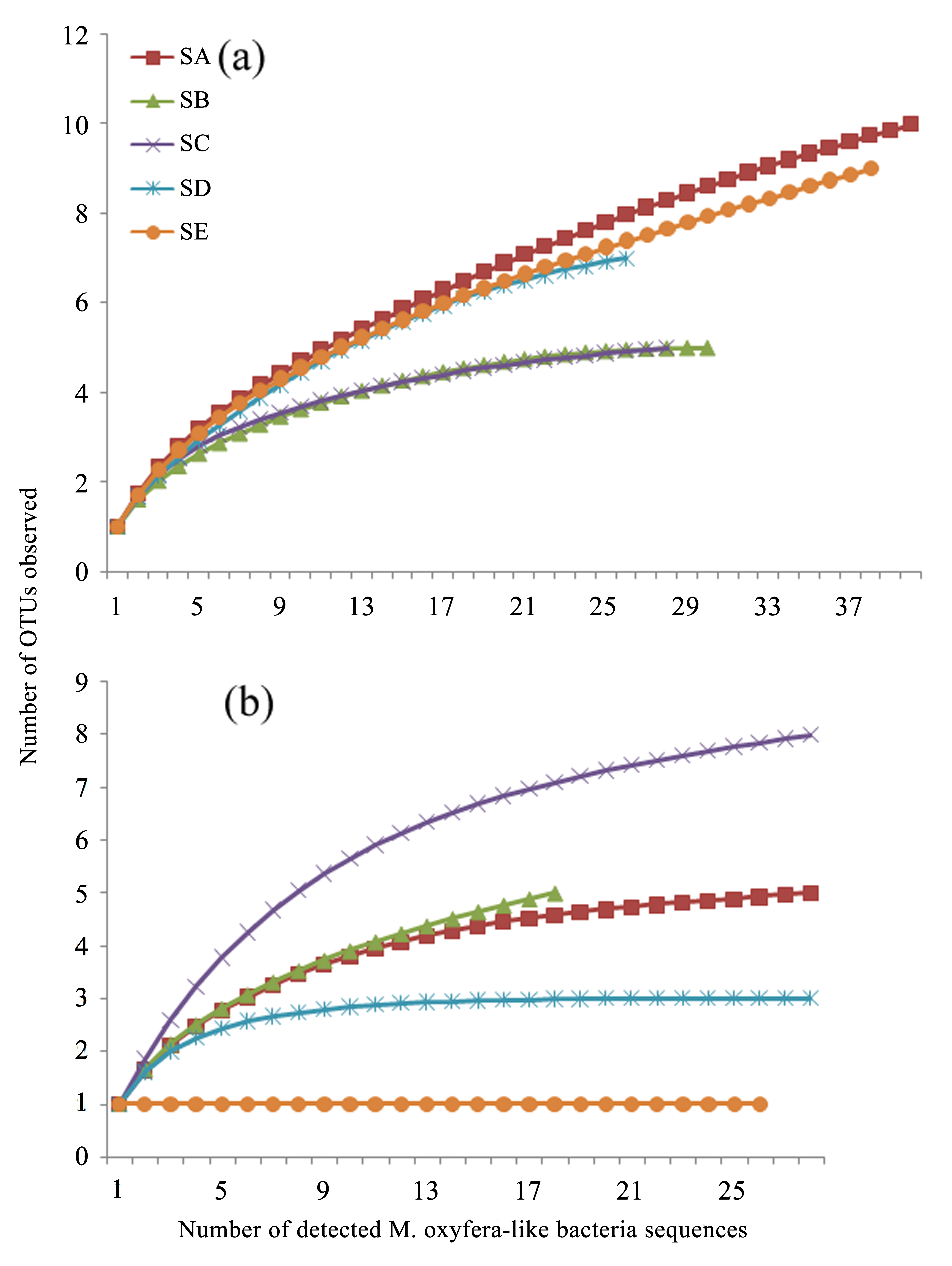

Supplement: S1 Fig — Mothur was used with 3% or 7% nucleotide sequence variation for OTU determination for 16S rRNA or pmoA gene, respectively. (TIF) [file pone.0137996.s001.tif]

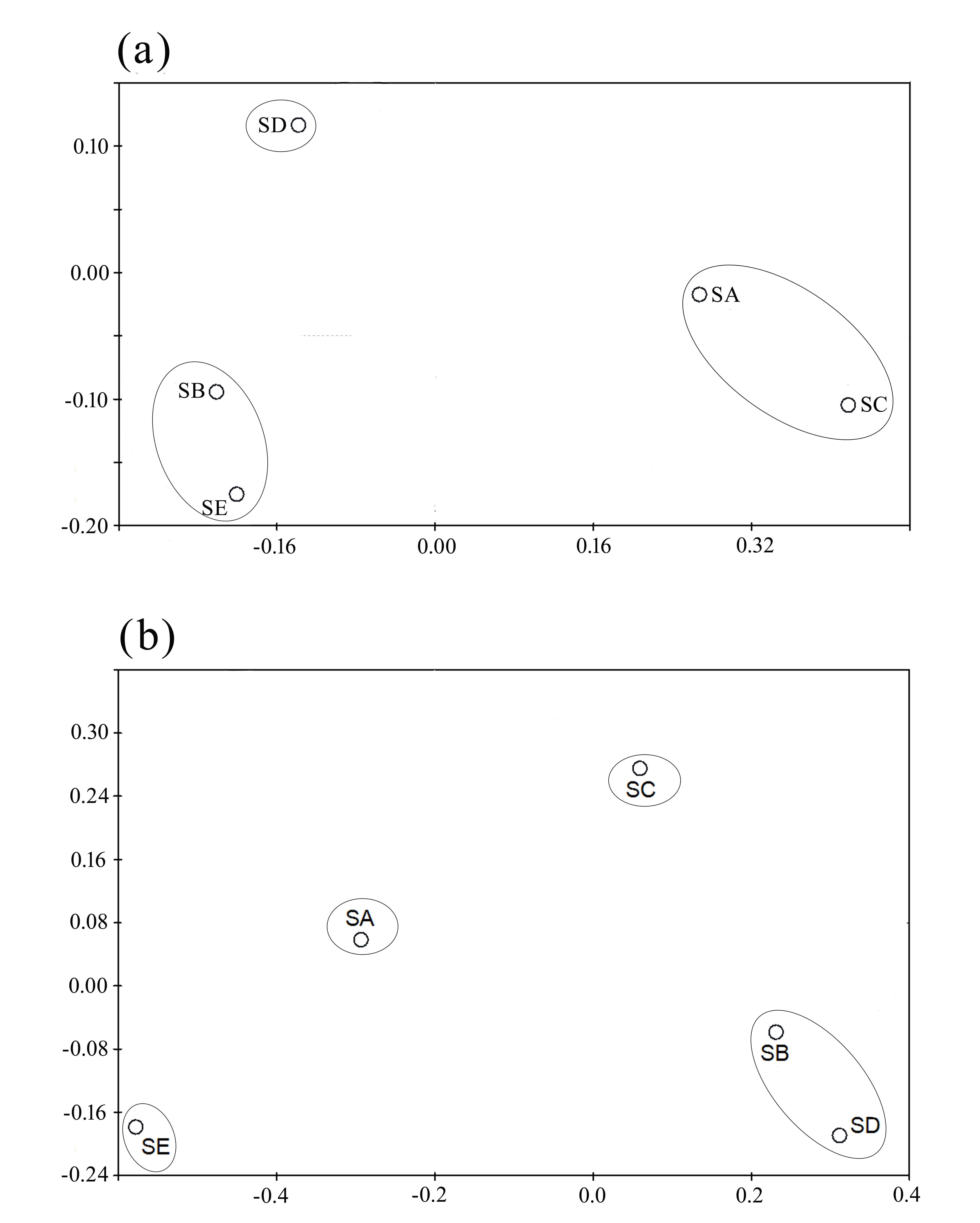

Supplement: S2 Fig — (TIF) [file pone.0137996.s002.tif]
