# Supplementary material for: Molecular Fingerprint and Dominant Environmental Factors of Nitrite-Dependent Anaerobic Methane-Oxidizing Bacteria in Sediments from the Yellow River Estuary, China
Source: PLoS One. 2015 Sep 14;10(9):e0137996. doi: 10.1371/journal.pone.0137996 (PMC4569144; doi:10.1371/journal.pone.0137996)
Supplement: S1 Table — (DOCX) [file pone.0137996.s003.docx]

**S1 Table.** Physicochemical characteristics of the sediment samples used in present study

| Sampling  site | Depth  (m) | pH | TN  (mg kg^-1^) | TP  (mg kg^-1^) | NH_4_^+^-N  (mg kg^-1^) | NO_3_^-^-N  (mg kg^-1^) | TOC  (mg kg^-1^) | BW-pH | BW-Sal  (‰) | BW-DO  (mg L^-1^) |
| --- | --- | --- | --- | --- | --- | --- | --- | --- | --- | --- |
| SA  SB  SC  SD  SE | 7.5  0.9  2.4  2.7  0.9 | 7.99  8.06  7.83  7.89  8.44 | 1359  1137  1391  816  869 | 164.61  171.82  100.22  110.75  168.48 | 19.46  7.08  27.74  21.47  12.58 | 0.90  0.61  0.67  0.23  0.35 | 4241  1305  5166  6053  1505 | 8.04  7.97  7.90  7.98  8.25 | 25.5  26.8  27.5  25.7  0.1 | 7.88  6.25  6.01  8.52  6.53 |

*Depth*: overlying water, *TN*: total nitrogen, *TP*: total phosphorus, *NH_4_^+^-N*: ammonium, *NO_3_^-^-N:* nitrate, *TOC*: total organic carbon, *BW*: bottom water, *Sal*: Salinity, *DO*: dissolved oxygen
