# Supplementary material for: Molecular Fingerprint and Dominant Environmental Factors of Nitrite-Dependent Anaerobic Methane-Oxidizing Bacteria in Sediments from the Yellow River Estuary, China
Source: PLoS One. 2015 Sep 14;10(9):e0137996. doi: 10.1371/journal.pone.0137996 (PMC4569144; doi:10.1371/journal.pone.0137996)
Supplement: S2 Table — (DOCX) [file pone.0137996.s004.docx]

**S2 Table.** The primers and thermal profiles used in this study

| Primer | Sequence (5́-3́) | Specificity | Position | Thermal profiles | References |
| --- | --- | --- | --- | --- | --- |
| 202F (PCR) | GACCAAAGGGGG  CGAGCG | NC10 phylum 16S | 193 | 4 min at 94 °C, followed by 35 cycles of 1 min at 94 °C, 1 min at 57 °C, 1.5 min at 72 °C, then 10 min at 72 °C | [1] |
| 1545R (PCR) | CAKAAAGGAGGT  GATCC | Bacteria 16S | 1529–1545 |  |  |
| qp1F ((q)PCR) | GGGCTTGACATCC  CACGAACCTG | n-damo 16S | 1001 | 4 min at 94 °C, followed by 35 cycles of 1 min at 94 °C, 1 min at 60 °C, 1 min at 72 °C, then 10 min at 72 °C (PCR, qp1F-qp2R) | [1] |
| qp2R (PCR) | CTCAGCGACTTCG  AGTACAG | n-damo 16S | 1481–1500 |  |  |
| qp1R (qPCR) | CGCCTTCCTCCAG  CTTGACGC | n-damo 16S | 1201 | 1 min at 95 °C, followed by 40cycles of 10 s at 95 °C, 10 s at 64 °C, 15 s at 72 °C (qPCR, qp1F-qp1R) | This study |
| A189_b (PCR) | GGNGACTGGGAC  TTCTGG | n-damo *pmoA* |  | 4 min at 94 °C, followed by 35 cycles of 1 min at 94 °C, 1 min at 50-60 °C, 1.5 min at 72 °C, then 10 min at 72 °C(PCR, A189_b-cmo682;cmo182-cmo568) | [2] |
| cmo682  (PCR) | AAAYCCGGCRAA  GAACGA | n-damo *pmoA* |  |  |  |
| cmo182  ((q)PCR) | TCACGTTGACGC  CGATCC | n-damo *pmoA* |  | 1 min at 95 °C, followed by 40 cycles of 10 s at 95 °C, 10 s at 56 °C, 25 s at 72 °C (qPCR, cmo182-cmo568) | [3] |
| cmo568  ((q)PCR) | GCACATACCCATC  CCCATC | n-damo *pmoA* |  |  |  |

**Reference**

1. Ettwig KF, van de Pas-Schoonen KT, Jetten MSM, Strous M. (2009) Enrichment and Molecular Detection of Denitrifying Methanotrophic Bacteria of the NC10 Phylum. Appl Environ Microbiol 75: 3656–62.

2. Luesken FA, Zhu BL, van Alen TA, Butler MK, Rodriguez Diaz M, Song B, et al. (2011) *pmoA* primers for detection of anaerobic methanotrophs. Appl Environ Microbiol 77: 3877–80.

3. Deutzmann JS, Schink B. (2011) Anaerobic Oxidation of Methane in Sediments of Lake Constance, an Oligotrophic Freshwater Lake. Appl Environ Microbiol 77: 4429–36.
